# Complete mitochondrial genome sequences and phylogenetic relationship of *Elysia ornata* (Swainson, 1840) (Mollusca, Gastropoda, Heterobranchia, Sacoglossa)

**DOI:** 10.1080/23802359.2016.1155427

**Published:** 2016-03-28

**Authors:** Mustafa Zafer Karagozlu, JinMo Sung, JeaHyun Lee, Taehyung Kwon, Chang-Bae Kim

**Affiliations:** a Department of Life Science, Sangmyung University, Seoul, Korea;; b Bionics Co., Ltd, Seoul, Korea;; c C&K Genomics, Seoul, Korea

**Keywords:** *Elysia ornata*, Gastropoda, mitochondrial genome, phylogenetic tree, Sacoglossa

## Abstract

The sacoglossans are small sea slugs in the Heterobranchia. In this study, mitochondrial genome of a sacoglossan sea slug, *Elysia ornata* (Swainson, 1840) is analyzed and phylogenetic tree of the species in the Heterobranchia is reconstructed based on amino acid sequences of mitochondrial protein coding genes. The phylogenetic relationship analysis shows that *E. ornata* belongs to the monophyletic Sacoglossa in the Heterobranchia and the Sacoglossa shows sister group relationship with a lineage including Anaspidea and Siphonarioidea. This is the fourth record for the mitochondrial genome of sacoglossan sea slugs.

The Sacoglossa is a highly specialized group which is included in the Heterobranchia but phylogenetic relationship of the Sacoglossa in the Heterobranchia is contradictory (Jörger et al. 2010; Medina et al. 2011) because the taxon sampling is still scarce in the Heterobranchia. *Elysia ornata* (Swainson, 1840) is recorded from the Pacific Ocean and Caribbean Sea. Krug et al. (2013) reported four Indo-Pacific candidate species in the *E. ornata* complex by examination of COI and nuclear H3 genes. In this study, complete mitochondrial genome of *E. ornata* which was collected from a subtidal area of Munsum Island (33°13′10″N, 126°30′49″E), Seogwipo, Jeju Island, Korea by scuba diving (Marine Biodiversity Institute of Korea Accession number MABIK MO00157621) was analyzed and the phylogenetic relationship of the species in the Heterobranchia was reconstructed based on amino acid sequences of the mitochondrial genes.

Mitochondrial genome length of *E. ornata* is 14 188 bp (GenBank accession number: KU365324). It is similar to mitochondrial genome of known sacoglossan sea slugs. The content of the mitochondrial genome is 13 protein coding genes, two ribosomal RNA genes and 22 tRNA genes. The gene order of the mitochondrial genome is also typical to the mitochondrial genomes of sacoglossan sea slug species. The phylogenetic relationship shows that *E. ornata* belongs to the monophyletic Sacoglossa (Figure 1). Besides the closest speciest to *E. ornata* among complete mitogenome known sacoglossan is *E. chlorotica*. The previous complete mitogenome based study (Medina et al. 2011) and molecular study using two mitochondrial and two nuclear genes (Jörger et al. 2010) showed that Sacoglossa has sister group relationship with Siphonarioidea. Our data also suggest that the Sacoglossa has sister group relationship with a lineage including Anaspidea and Siphonarioidea. In the present phylogeny, Anaspidea, Siphonarioidea, Sacoglossa, Acteonoidea, Nudiranchia, Pyramidelloidea, Amphiboloidea, Ellobioidea, Systellommatophora and Hygrophila in the Heterobranchia are monophyletic groups. The present study will provide data for taxonomy of the Sacoglossa with comprehensive taxon sampling.

**Figure 1.**
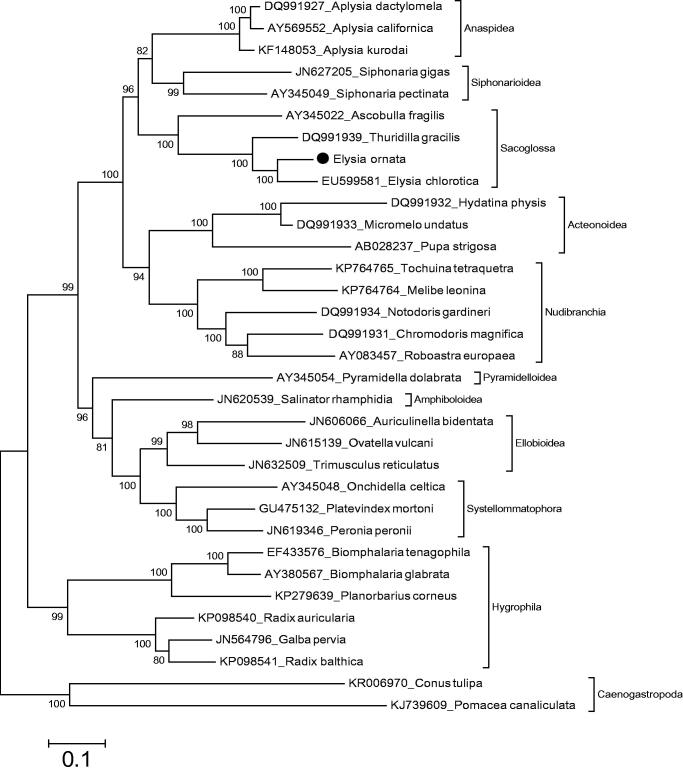
Phylogenetic relationship of *Elysia ornata* in the Heterobranchia. The specimen was preserved in 97% ethanol and DNA was extracted from foot. Paired end reads (2 × 300 bp) were generated from a mitochondrial enriched genomic library. The reads (616 664 400 bp) were assembled and annotated by using MITObim (Hahn et al. 2013) and MITOS (Bernt et al. 2013), respectively. The complete mitochondrial genomes of the Heterobranchia species were retrieved from the GenBank for the reconstruction of phylogenetic relationship of *E. ornata*. Phylogenetic tree based on concatenated amino acid sequences from 12 protein coding mitochondrial genes was reconstructed by MEGA (Kumar et al. 1993) with maximum likelihood analyses based on mtREV with Freqs (+F) model. ATP8 gene was excluded from the analysis. Bootstrap method used 1000 replicates to know statistical support. Two species of the Caenogastropoda were used as outgroups.
